# Space-time analysis of ovarian cancer mortality rates by age groups in spanish provinces (1989–2015)

**DOI:** 10.1186/s12889-020-09267-3

**Published:** 2020-08-17

**Authors:** Paula Camelia Trandafir, Aritz Adin, María Dolores Ugarte

**Affiliations:** 1 Department of Statistics, Computer Science, and Mathematics, Public University of Navarre, Campus de Arrosadia, Pamplona, 31006 Spain; 2 INAMAT, Public University of Navarre, Campus de Arrosadia, Pamplona, 31006 Spain

**Keywords:** Age-space-time models, Disease mapping, INLA, Ovarian cancer mortality, Smoothing

## Abstract

**Background:**

Ovarian cancer is a silent and largely asymptomatic cancer, leading to late diagnosis and worse prognosis. The late-stage detection and low survival rates, makes the study of the space-time evolution of ovarian cancer particularly relevant. In addition, research of this cancer in small areas (like provinces or counties) is still scarce.

**Methods:**

The study presented here covers all ovarian cancer deaths for women over 50 years of age in the provinces of Spain during the period 1989-2015. Spatio-temporal models have been fitted to smooth ovarian cancer mortality rates in age groups [50,60), [60,70), [70,80), and [80,+), borrowing information from spatial and temporal neighbours. Model fitting and inference has been carried out using the Integrated Nested Laplace Approximation (INLA) technique.

**Results:**

Large differences in ovarian cancer mortality among the age groups have been found, with higher mortality rates in the older age groups. Striking differences are observed between northern and southern Spain. The global temporal trends (by age group) reveal that the evolution of ovarian cancer over the whole of Spain has remained nearly constant since the early 2000s.

**Conclusion:**

Differences in ovarian cancer mortality exist among the Spanish provinces, years, and age groups. As the exact causes of ovarian cancer remain unknown, spatio-temporal analyses by age groups are essential to discover inequalities in ovarian cancer mortality. Women over 60 years of age should be the focus of follow-up studies as the mortality rates remain constant since 2002. High-mortality provinces should also be monitored to look for specific risk factors.

## Background

The number and scientific impact of publications on ovarian cancer are continuously increasing [1]. Not in vain, ovarian cancer is the eighth most common cancer in women and the 18^th^ most frequent overall, with nearly 300,000 new cases worldwide in 2018 [2]. Around 50% of cases are concentrated in developed countries, where ovarian cancer is the most lethal gynecological tumor. The highest incidences are found in northern and eastern Europe (Austria, the Czech Republic, Germany, Ireland, Latvia, Lithuania, the Nordic countries, Slovakia, and the UK) and the United States [3], whereas in Africa and Asia this tumor is virtually non-existent. This pattern is attributed mostly to the low birth rates found in developed countries [4].

In the past 20 years, the overall number of tumors has undergone a constant increase in Spain. This is due not only to the population growth, but also to the increased life expectancy and the use of early detection techniques. In 2017, the estimated number of new cases of ovarian cancer was 3142, representing 5.1*%* of all female cancers, being the fifth cause of cancer deaths in women after lung, breast, colon, and uterine tumors [5, 6]. The age-standardized incidence rate, calculated using the direct method and the world standard population, is 7.4 per 100,000 women, which may be considered high. As for its temporal evolution, there was a slight decline between 1997 and 2003, but since then, mortality rate starts to increase slowly, but constantly. In addition, there is a great deal of variability among the Spanish provinces; for example, during the period 2003–2007, one finds a rate of 6.7 per 100,000 women in the Canary Islands and a rate of 10.0 per 100,000 women in Cuenca [7].

Ovarian cancer is a disease affecting mostly older postmenopausal women, with more than 80% of cases being diagnosed in women over 50 [8]. According to medical experts, it is a silent and mostly asymptomatic cancer, a circumstance that leads to late diagnosis and worse prognosis [9]. Furthermore, in the cases where symptoms do appear, these may be confused with digestive problems (bloating, early satiety, abdominal and/or pelvic pain) or benign gynecological alterations such as endometriosis or polycystic ovary syndrome. To date, no method for early detection is available. This is reflected in the fact that up to 75% of cases are detected in the advanced stages of the disease [5].

The etiology of ovarian cancer is poorly understood. However, several factors associated with an increased risk of ovarian cancer have been identified: age, number of ovulations (early menarche, infertility, low parity), the use of hormone replacement therapy (HRT) [10], obesity, physical inactivity, a family history of breast and ovarian cancer, including BRCA1 and BRCA2 gene mutations [11], and past and current smoking [12]. Associations between exposure to asbestos in the workplace or at home and ovarian cancer have also been found [13]. Further research is needed to corroborate this finding in Spain as most jobs with a high exposure to asbestos are predominantly male-dominated, e.g. mining, milling or shipyard work. Nevertheless, a study of asbestos exposure among Italian women [14] found that the main factor was second-hand contact due to occupationally exposed relatives, for example from soiled work clothes brought home. As of this writing, however, there is no documented registry for asbestos exposure in the workplace or at home in Spain. Some epidemiological studies have detected an increased risk of ovarian cancer in women with less exposure to sunlight and consequently with low levels of vitamin D. In particular, the higher the latitude, the less overall accumulated sunlight, and the higher the incidence of ovarian cancer [15]. Some protective factors against ovarian cancer have also been identified such as multiparity, oral contraceptives, and tubal ligation or hysterectomy [16].

The 5-year age-standardised relative survival in Spain (2000-2007) is estimated at less than 36.8*%*, similar to the European average of 37.6*%* [17]. As mentioned above, ovarian tumors are the eighth cause of cancer deaths among women worldwide, with 125,000 deceases registered in 2002 (4.3*%* overall among malign tumors) [17]. In 2016, about 1,960 women died yearly in Spain from ovarian cancer, representing 4.4*%* of all cancer deaths and 1.7*%* of all deaths among women [18].

The mean age of decease from ovarian cancer in Spain is 67.7 years. Cabanes et al. (2009) [19] analyzed the age-adjusted mortality trends of ovarian cancer in Spain for the period 1980–2006. Ovarian cancer caused 36,157 deaths in this period, with rates in women over 50 showing a ten-fold increase versus those in younger women. In women under 50, rates increased 1.6*%* per year until 1995, and afterwards started to drop at a rate of −1.4*%* per year. In the age group 50–64, mortality rates significantly increased 4.4*%* annually up to 1998 and became stable thereafter. In women 65 and older, mortality rates increased 5.8*%* annually up to the year 2000, and decreased 2.0*%* per year after.

The disproportionate impact on older women, together with the aforementioned concerns regarding late-stage detection and low survival rates, makes the study of the space-time evolution of ovarian cancer particularly relevant. In addition, it is important to mention that age groups are not equally affected by ovarian cancer mortality, and then, it is necessary not just to standardize by age, but to analyze the different age groups. Hence, the main goal in this paper is to study the temporal evolution of the geographical patterns of ovarian cancer mortality rates in four age groups of women aged 50 years or more.

## Methods

### Data source

The study presented here covers all ovarian cancer deaths (code C56 of the 10th edition of the International Classification of Diseases [20]) for women over 50 years of age in the 50 provinces of Spain (excluding the autonomous cities of Ceuta and Melilla), recorded throughout the period 1989–2015 by the Spanish Statistical Office.

### Statistical analysis

A Bayesian hierarchical spatio-temporal model is used to estimate rates [21]. The model is briefly described in what follows for better interpretation of results.

Spain is divided into *S*=50 provinces indexed by *i*=1,…,*S*, and data are available for T=27 time periods (corresponding to years 1989–2015) labeled as *t*=1,…,*T*. For each age group, let *N*_*it*_ represent the population at risk for region *i* and time period *t*. Then, conditional on the mortality rates *r*_*it*_, the number of ovarian cancer deaths *O*_*it*_ is assumed to follow a Poisson distribution with mean *μ*_*it*_=*N*_*it*_*r*_*it*_, that is,

$$\begin{array}{@{}rcl@{}} \begin{array}{rcl} O_{it}|r_{it} & \sim & \text{Poisson}(\mu_{it}=N_{it}r_{it})\\ \log \mu_{it} & = & \log N_{it} + \log r_{it}, \end{array} \end{array} $$

where the log-rate is modelled as

1 $$ \log r_{it} = \alpha+\xi_{i}+\gamma_{t}+\delta_{it}.   $$

Here, *α* denotes the logarithm of the overall rate, *ξ*_*i*_ and *γ*_*t*_ are the main spatial and temporal effects respectively, and *δ*_*it*_ corresponds to the space-time interaction effects. Since each of these components are supposed to be Gaussian Markov random fields (GMRF) [22], the integrated nested Laplace approximation (INLA)[23] technique has been used for model fitting and inference. Specifically, the Leroux et al. [24] CAR prior distribution has been considered for the spatial random effects and a first-order random walk prior distribution for the temporal random effects. In addition, the four different types of interaction introduced by Knorr-Held [25] have been considered for the spatio-temporal random effects. These interactions allow the space-time effects to be completely independent (Type I interaction), structured in time but not in space (Type II interaction), structured in space but not in time (Type III interaction), or completely structured in space and time (Type IV interaction).

All the computations have been done using the interactive web application SSTCDapp [26], which can be found at https://emi-sstcdapp.unavarra.es/Login/. This application provides a user interface for the analysis of spatio-temporal areal count data allowing to fit a wide variety of space-time models using the INLA estimation technique. In addition, the application provides different model selection criteria. In this paper, the model with the lowest value of the Deviance Information Criterion (DIC) [27] has been selected. For further details about model specification, prior distribution of the hyperparameters, identifiability constraints, and additional model selection criteria see for example Adin et al. [26] and the references therein.

## Results

**Fig. 1:**
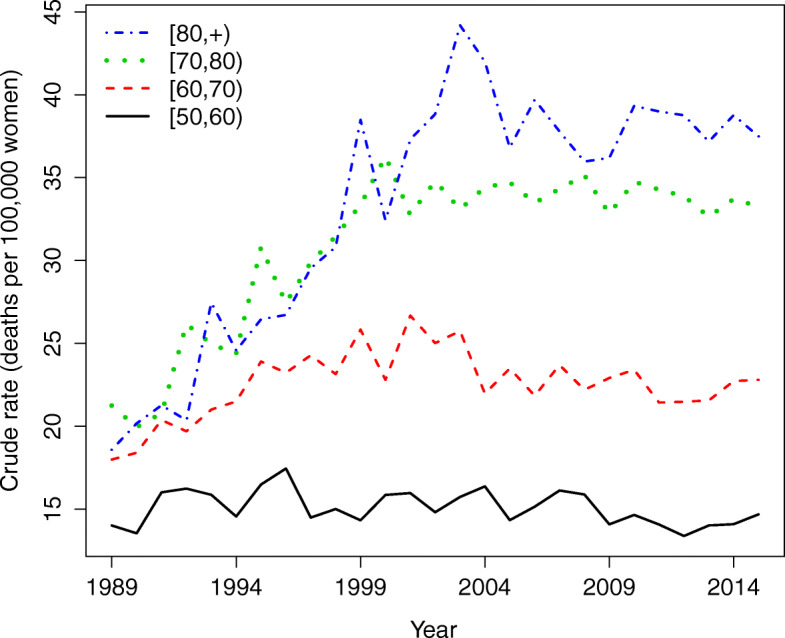
Temporal trends by age groups. Temporal trend of the ovarian cancer mortality (crude) rates by age-groups

A total of 28,350 ovarian cancer deaths were registered in the population of Spanish women over 50 years of age during the period 1989–2015. Since ovarian cancer is mainly related to the onset of menopause, the age groups we are considering in this paper are [50,60),[60,70),[70,80), and [80,+). A brief summary of observed cases and mortality rates (per 100,000 women) by age groups, province and year is shown in Table 1. Clear differences are observed in the mean and median mortality rates among the youngest and oldest age groups, with values ranging from 15 cases per 100,000 women up to 32 cases per 100,000 women (approximately) respectively. Figure 1 displays the global temporal trend of crude rates by age group. Here the different behaviour of the age groups is even more evident. A pronounced slope from the last decade of the twentieth century to the beginning of the twenty-first century is observed in the older age groups.

**Table 1:** Descriptive statistics of observed cases and crude mortality rates per 100,000 women disaggregated by age groups, province, and year. Min: minimum; *Q*_1_: first quartile; *Q*_3_: third quartile; Max: maximum

	Age group	Min	*Q*_1_	Median	Mean	*Q*_3_	Max
Observed cases	[50,60)	0.00	2.00	5.00	7.00	8.00	63.00
	[60,70)	0.00	3.00	5.00	9.20	10.00	79.00
	[70,80)	0.00	3.00	6.00	9.10	11.00	81.00
	[80,+)	0.00	1.00	2.00	3.80	5.00	49.00
Crude rates	[50,60)	0.00	10.17	14.73	15.38	19.44	65.46
	[60,70)	0.00	15.80	21.84	22.61	28.48	93.25
	[70,80)	0.00	20.91	30.46	30.66	39.49	139.39
	[80,+)	0.00	15.83	30.96	32.27	46.25	158.38

**Fig. 2:**
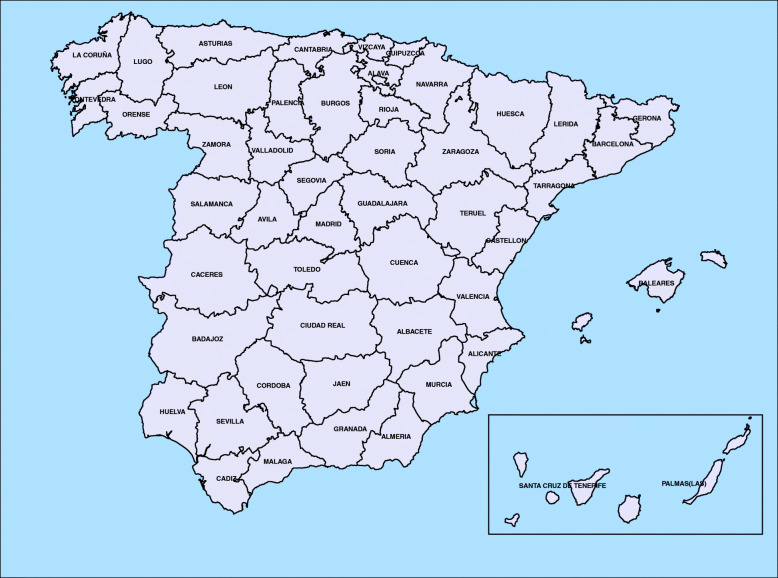
Administrative division of Spain. Map with the administrative division of Spain showing provinces. Source: map was generated by the authors using the library tmap [29] from the R statistical software version 3.6.2 [30]. No licenses are required to use or publish

Model (1) was fitted to smooth spatio-temporal rates in each age group. The interaction considered in the model was chosen on the basis of the DIC values for each sub-group of age-class. The DIC pointed toward a Type IV interaction for age groups [50,60),[70,80), and [80,+) whereas a Type II interaction was selected for the age group [60,70). To make the different terms in all the models comparable, a decomposition of the estimated log-rates was computed by defining posterior spatial ($\xi _{i}^{*}$), temporal ($\gamma _{t}^{*}$) and spatio-temporal ($\delta _{it}^{*}$) patterns (see Adin et al., [28]), so that $\log r_{it}=\alpha ^{*}+\xi _{i}^{*}+\gamma _{t}^{*}+\delta _{it}^{*}$. Note that exp(*α*^∗^) represents the overall mortality rate for the whole of Spain during the period 1989–2015. In order to facilitate interpretation of the results, a map of Spain showing its provinces is given in Fig. 2.

**Fig. 3:**
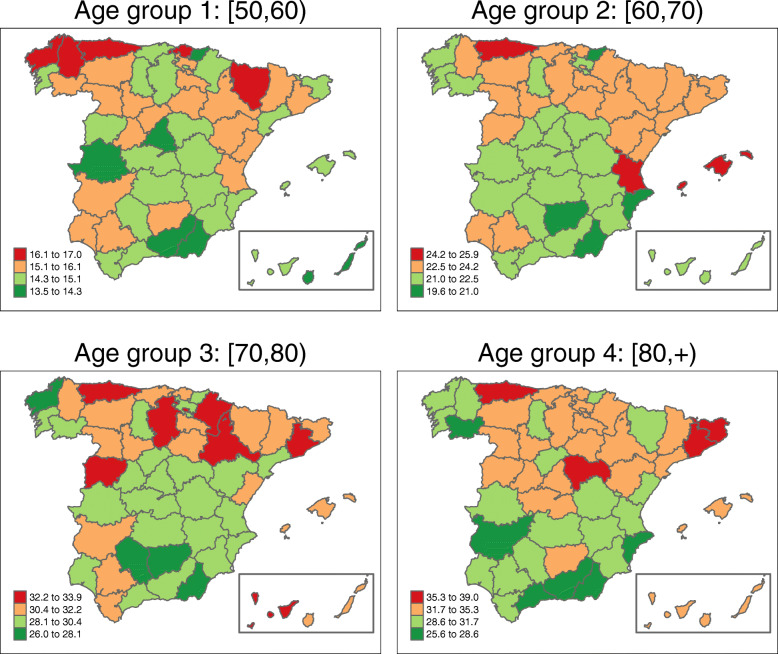
Province-specific mortality rates’ estimates by age-groups. Posterior mean estimates of province-specific mortality rates $\exp (\alpha ^{*}+\xi _{i}^{*})$. Source: maps were generated by the authors using the library tmap [29] from the R statistical software version 3.6.2 [30]. No licenses are required to use or publish

**Fig. 4:**
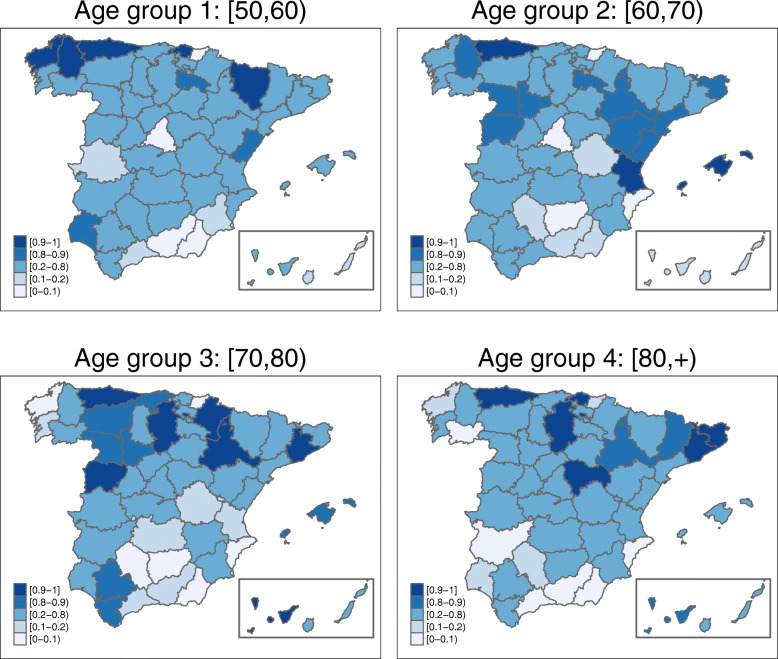
Province-specific posterior exceedance probabilities by age-groups. Posterior exceedance probabilities of each province in comparison with the Spanish overall rate $P(\exp (\alpha ^{*}+\xi _{i}^{*})>\exp (\alpha ^{*}) | \mathbf {O})$. Source: maps were generated by the authors using the library tmap [29] from the R statistical software version 3.6.2 [30]. No licenses are required to use or publish

Figure 3 shows the map with the posterior mean estimates of province-specific mortality rates, i.e., $\exp (\alpha ^{*}+\xi _{i}^{*})$. Posterior exceedance probabilities of this province-specific rate being greater than the overall Spanish rate have also been computed (see Fig. 4).

The estimated spatial pattern draws attention to Asturias as a high ovarian cancer mortality rate province for all age groups. In the age group [50,60), the highest spatial rates are found in the northwestern provinces (Asturias, Lugo, and La Coruña) but also in Vizcaya and Huesca (over 16 cases per 100,000 women). In age group [60,70) the regions with the highest estimated rates are Asturias, the Balearic Islands, and Valencia (with an estimated rate of over 24 cases per 100, 000 women). In the third age group ([70,80)), the highest rates are located in the central-northern areas (with Salamanca and Asturias leading the ranking), and in the Canary Islands (Tenerife province) (all of them with more than 33 cases per 100,000 women). The oldest age group ([80,+)) exhibits high rate areas in Asturias, Barcelona, Gerona, and Guadalajara, with a rate of over 35 cases per 100,000 women. All of these high-rate provinces have a rate significantly higher than the overall Spanish rate in their respective age groups (see Fig. 4).

In general, northern Spain has greater ovarian cancer mortality rates compared to the southern regions. The lowest rates are found in Guipúzcoa for age groups [50,60) and [60, 70), and in Almería for age groups [70,80) and [80, +). The northwestern province of La Coruña shows a surprising behaviour, with high rates in the age group [50,60) but one of the lowest rates in the age group [70,80).

**Fig. 5:**
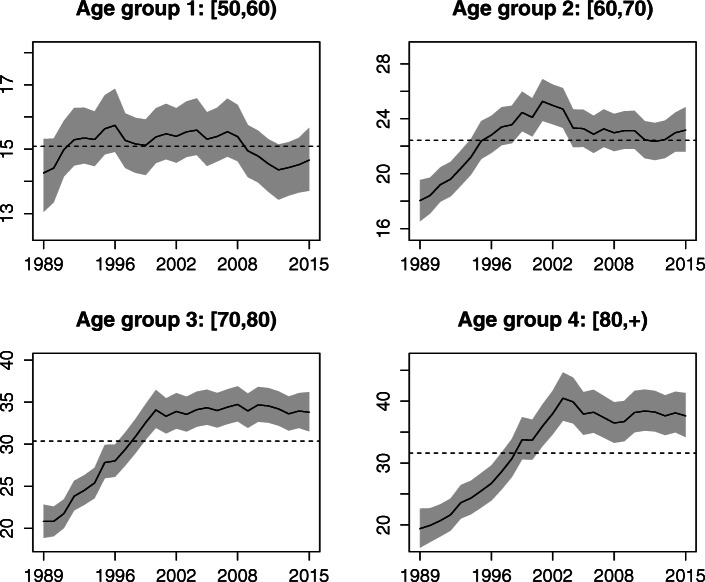
Temporal trends’ estimates by age-groups for the whole of Spain. Posterior mean estimates of year-specific mortality rates ($\exp (\alpha ^{*}+\gamma _{t}^{*})$) and 95% credible intervals. Dotted lines provide the estimated rate in each age group in the whole period in Spain (exp(*α*^∗^))

The estimated global temporal pattern $\exp (\alpha ^{*}+\gamma _{t}^{*})$ is visualized in Fig. 5. Rates seem to have decreased during the last few years (from 2008 to 2013) in age group [50, 60) but have remained nearly constant in the other three age groups, which experienced a sharp increase in rates from 1989 up to the beginning of the twenty-first century approximately.

**Fig. 6:**
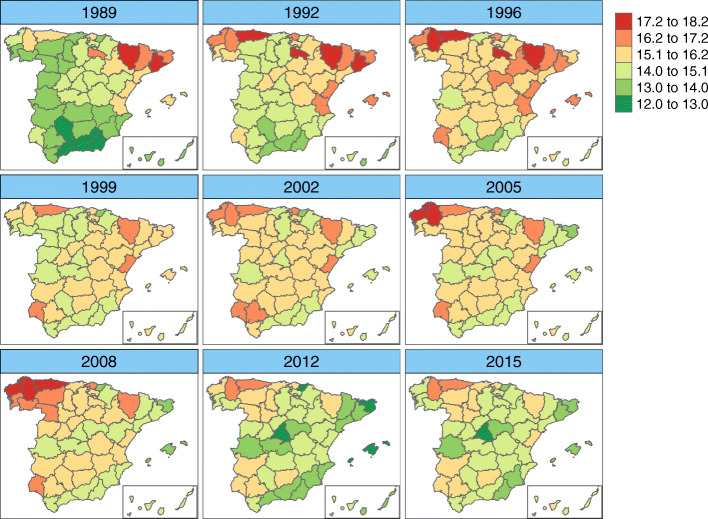
Mortality rates’ estimates for age group [50,60). Posterior mean estimates of mortality rates *r*_*it*_ for age group [50,60). Source: maps were generated by the authors using the library tmap [29] from the R statistical software version 3.6.2 [30]. No licenses are required to use or publish

**Fig. 7:**
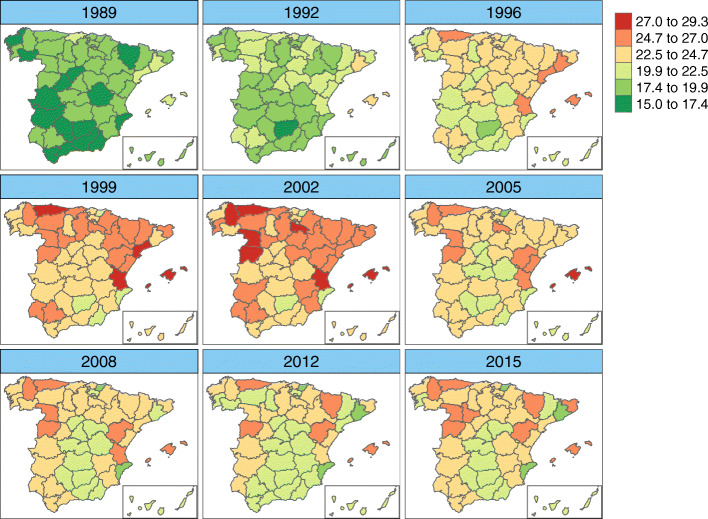
Mortality rates’ estimates for age group [60,70). Posterior mean estimates of mortality rates *r*_*it*_ for age group [60,70). Source: maps were generated by the authors using the library tmap [29] from the R statistical software version 3.6.2 [30]. No licenses are required to use or publish

**Fig. 8:**
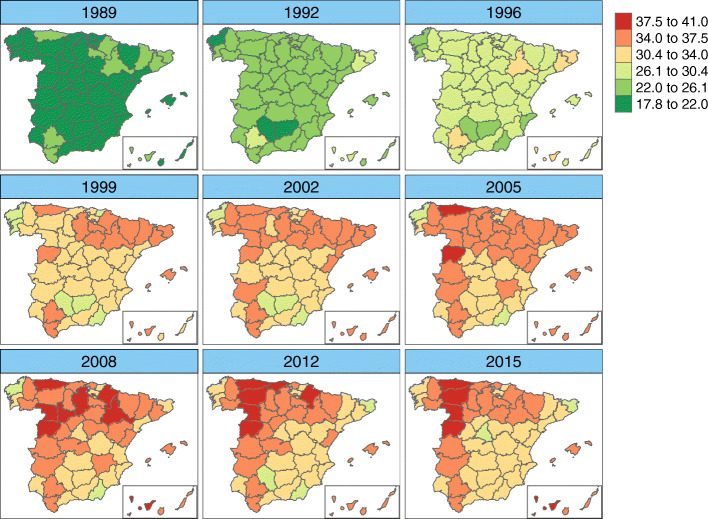
Mortality rates’ estimates for age group [70,80). Posterior mean estimates of mortality rates *r*_*it*_ for age group [70,80). Source: maps were generated by the authors using the library tmap [29] from the R statistical software version 3.6.2 [30]. No licenses are required to use or publish

**Fig. 9:**
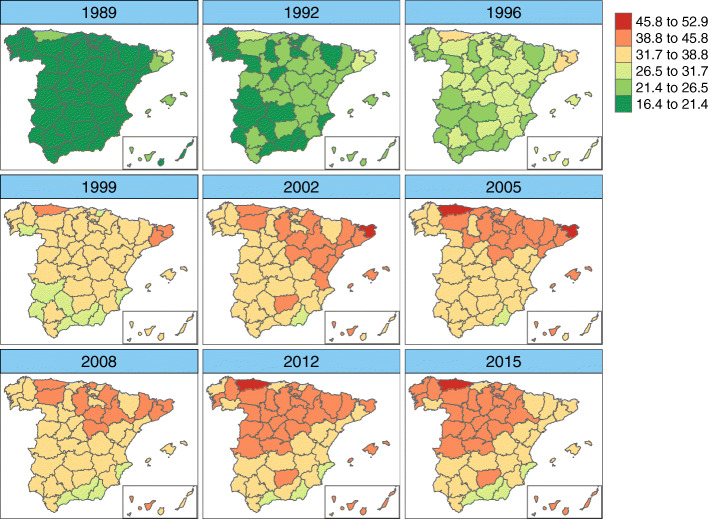
Mortality rates’ estimates for age group [80,+). Posterior mean estimates of mortality rates *r*_*it*_ for age group [80,+). Source: maps were generated by the authors using the library tmap [29] from the R statistical software version 3.6.2 [30]. No licenses are required to use or publish

Figures 6, 7, 8, and 9 display maps showing the spatio-temporal evolution of ovarian cancer mortality rates for each Spanish province for the period 1989–2015 (divided into intervals of 3–4 years) for age groups [50, 60), [60, 70), [70, 80), and [80,+), respectively. Specifically, these maps represent the posterior mean of $r_{it}=\exp (\alpha ^{*}+\xi _{i}^{*}+\gamma _{t}^{*}+\delta _{it}^{*})$. The corresponding maps of probabilities (showing the probability that a particular rate in a given province and year is greater than the Spanish rate during that period) are not shown here to conserve space. However, we have classified a province as having a rate significantly greater than the Spanish rate if this probability is greater than 0.8.

For age group [50,60), we find that the mortality rate for the period 1989–1999 was highest in the northeastern region, with the province of Huesca having a significant rate that lasted until 2008. In the latter years of the period studied, only Asturias showed a significant rate. Within this age group, the percentage of the rate’s variability explained by the spatio-temporal term was 29.4%, implying that the specific temporal evolution of each province is rather high in this age group.

In age group 2 ([60, 70)), rates exhibit a greater variability among provinces (between 15 cases per 100,000 women and about 29 cases per 100,000 women). The highest rates occurred in the late 1990s and early 2000s, mainly in the northern half of Spain and in the Balearic Islands. Asturias, Lugo, Salamanca, Valladolid, Huesca, Teruel, Gerona, and the Balearic islands show significantly high rates at the end of the period.

For women between 70 and 80 years of age, the lowest rates are found at the beginning of the study period, with significantly low rates. The highest significant rates are located mainly in the northern and western parts of Spain and in the Canary and Balearic islands in the period 2005–2015. The provinces with the lowest rates at the end of the period are Gerona and Madrid.

Women over 80 years of age show significantly low rates between 1989–1996. Again, Asturias shows the highest mortality rate at the end of the period. Some provinces located in southern Spain, mainly along the coastline, show significantly low rates.

**Fig. 10:**
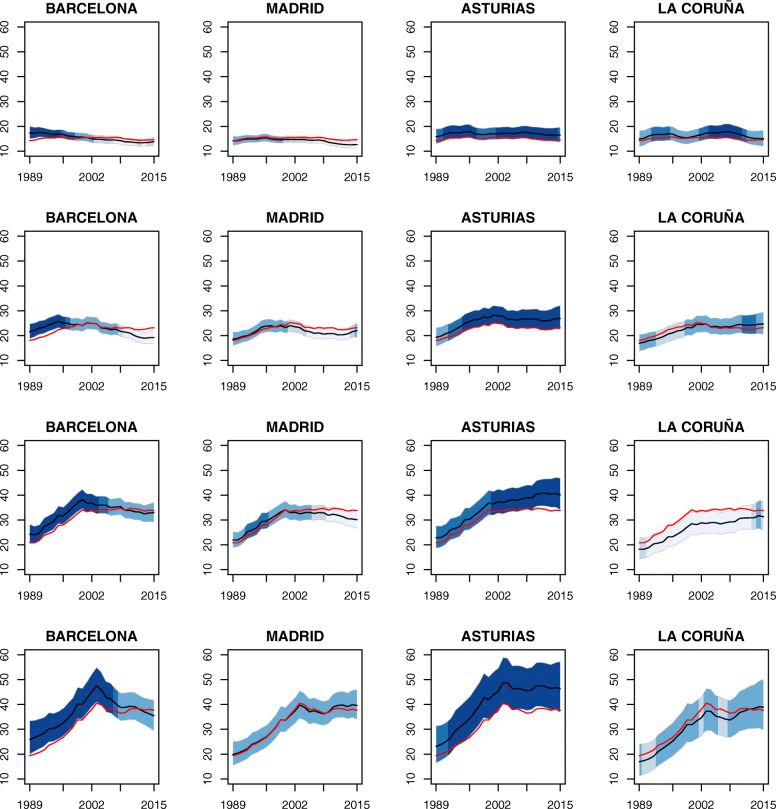
Temporal evolution of ovarian cancer mortality rates’ estimates for some selected provinces: Barcelona, Madrid, Asturias and La Coruña. Temporal evolutions of the posterior mean estimates of mortality rates *r*_*it*_ for some selected provinces and 95% two-sided credible intervals for age groups [50,60) (first row), [60,70) (second row), [70,80) (third row) and [80,+) (fourth row). The colors used in the bands are associated to the posterior exceedance probabilities of each province in time *t* in comparison with the temporal pattern of Spain, that is, $P(r_{it} > exp(\alpha ^{*} + \gamma _{t}^{*}) | \mathbf {O})$, represented with a red line in the graphs

The temporal evolution of the rates for some selected provinces are plotted in Fig. 10. The colors used in the bands are associated to the posterior exceedance probabilities of each province at year *t*, in comparison with the temporal pattern for the whole of Spain in that year, namely $P(r_{it} > \alpha ^{*} + \gamma _{t}^{*} | \mathbf {O}).$ In the case of Barcelona, the probability that the mortality rate lies above the Spanish rate reaches a maximum between 1989–1996 for age groups [50, 60) and [60,70) and between 1989–2003 for age groups [70,80) and [80, +). Madrid behaves similar to Spain in all age groups. In Asturias, the probability is quite high for all age groups, with a significant rising trend for age group [70,80) from 2002 onwards. La Coruña shows significantly low rates during the whole study period for the age group [70,80), whereas this province’s behaviour for women over 80 years of age is similar to the Spanish rate.

## Discussion

The ovaries are one of the cancer sites where known risk factors are not enough to explain all the cases, and thus spatio-temporal analyses provide additional important information allowing for the examination of the spatial, temporal, and spatio-temporal mortality patterns. As the age groups are not equally affected by ovarian cancer mortality, it is necessary not just to standardize by age, but also to analyze the different age groups.

Results show large differences in mortality among the age groups, with higher mortality rates in the older age groups. Indeed, the last age groups (women of more than 70 years of age) double the rate of women between 50 and 60 (more than 30 cases in the last age groups vs. 15.1 cases per 100,000 women on average in age group [50,60)). This can be explained by poor survival rates of gynaecological cancers in the elderly, which in turn is influenced by late diagnoses and the failure of treatments due to comorbidity [31, 32].

The global temporal trends (by age group) reveal that the evolution of ovarian cancer over the whole of Spain has remained nearly constant since the early 2000s, particularly for women aged 60 years or more, after a sharp increase during the period 1989–2002 (approximately). The stabilization of the rates may be due to an increased concern among women regarding their personal health (in particular among older women) that led them to be tested more frequently, access to better information via mass media and the internet, and the increasing effectiveness of cancer treatments. In the nineties, access to information via the internet was more limited and health care was possibly less advanced, which could explain in part the sharp increase in mortality rates in the first half of the period. Mortality in women between 50 and 60 years of age shows a slight decrease since 2008 until nearly 2013, although this decrease does not seem to be significant with respect to the average mortality in this age group. It seems that in fact, during the last two years of the study period, rates are starting to rise slightly, but once again, this trend is not significant as yet.

The global geographical patterns show, in general, that the North has higher rates than the South, a situation similar to that observed overall in Europe [3]. The variability observed in all age groups between northern and southern Spain remains unknown, although in general, women in the South are prone to marry earlier and have more children on average [33]. Interestingly, Asturias, the province with the highest rates in all age groups, is one of the Spanish provinces with the lowest average number of children. The differences observed between the North and the South could also be explained taking into account the relationship between exposure to sunlight (pro-vitamin D) and ovarian cancer. Although Spain is in general a sunny country, there is a great variability between the North and South in terms of average daily hours of sunlight. For example, Bilbao in the north receives about 1500 hours of sunlight per year while Sevilla in the south receives 3800 [34]. Thus, the observed increasing trend with latitude might be at least partly explained by a cumulative exposure to sunlight.

Heterogeneity among the provinces regarding ovarian cancer mortality rates can be elucidated at least in part by a heterogeneous distribution of other risk factors. There are differences among provinces in the age at which women have their first period (menarche), the average age of first childbirth, and the total number of children [35–37]. The average fecundity rate in Spain has been markedly declining; in particular, the areas registering the lowest fertility rates were the Basque Country, Asturias, Navarre, and Aragón [19, 36]. We should also point out that the age of first birthing is closely related to socioeconomic development, and is steadily increasing. Navarre and the Basque Country are the regions of Spain where women delayed childbearing the longest [37]. Additionally, hormonal replacement therapy has proven to have an important influence on the appearance of ovarian cancer in postmenopausal women [38–41]. However, its use in Spain has been very limited [42].

Another risk factor is the presence of a family history of the disease [43]. Hereditary ovarian cancer syndrome presenting a mutation in BRCA genes is important. Between 6–15% of ovarian cancer cases are linked to BCRA mutation. Women who are carriers of the BCRA1 mutation have a 39% chance of suffering ovarian cancer before age 70 [44]. In Spain, the accumulated risk of developing ovarian cancer before age 70 has been estimated at 22% (95% CI, 0–40%) in carriers of a mutation in BCRA1 and 18% (95% CI, 0–35%) in carriers of a mutation in BRCA2 [44]. The prevalence of BRCA1 and BRCA2 mutation in Spain is heterogeneous and varies according to geographical origin. Moreover, Blay et al. [45], showed that the BRCA1 and BRCA2 spectrum of mutations in Asturias was largely different from other areas of Spain. This could also explain in part the high mortality rates found in this province. Diez et al. [46], studying a large group of Spanish patients, showed that there is only a slight difference between the percentages of deleterious mutations in BRCA1 and BRCA2 genes (53% and 47%, respectively). However, some variation due to geographic origin is present, with a higher proportion of BRCA1 in families from the northwestern part of Spain. According to Vega et al. [47], the differences found in Galicia could be due to founder effects.

Ovarian cancer is also linked to lifestyle habits, tobacco and alcohol consumption. The smoking habit is a risk factor for epithelial ovarian cancer with an odds ratio of 2.98 (95% CI [1.15−7.73]) [12]. Differences in alcohol and tobacco consumption can be found among Spanish provinces [48].

All in all, to better understand the etiology of the disease and to better determine the effect of risk factors on the Spanish female population, it would have been helpful to have the medical and workplace histories of all the women participating in this study. This is the main limitation of the current work. On the other hand, as there is a lack of scientific studies analyzing the association between ovarian cancer mortality rates and risk factors in the domains analyzed here (age groups, provinces, and years), spatio-temporal analyses by age groups are essential to discover inequalities in ovarian cancer mortality, to detect provinces with high risks in each age group, and to keep track of how the rates are evolving with time.

## Conclusions

Differences in ovarian cancer mortality exist among the Spanish provinces, years, and age groups. As the exact causes of ovarian cancer remain unknown, spatio-temporal analyses by age groups are very useful to look for potential risk factors associated to the observed geographical patterns and to allocate funds among Spanish regions. For future clinical and epidemiological practice, we recommend to follow-up women over 60 years as the mortality rates remain constant since 2002. High-mortality provinces should also be monitored to look more closely for specific risk factors. Some risk factors for ovarian cancer, like getting old or having a family history, cannot be changed. However, women may slightly decrease their risk by avoiding other risk factors, for example, maintaining a healthy weight, avoiding tobacco and alcohol consumption or not receiving hormone replacement therapy after menopause.

## Data Availability

Data have been provided by the Spanish Statistical Institute at municipality level (under a contract) and aggregated later. Up on reasonable request, the corresponding author will make the datasets available. Mortality data from cancer and other causes from 1975 by sex and province is available in the Interactive Epidemiological Information System (ARIADNA, http://ariadna.cne.isciii.es/evindex.html) of the Spanish National Center for Epidemiology.

## Abbreviations

AEI: Spanish Research Agency
BRCA: Breast Cancer
DIC: Deviance Information Criterion
UE: European Union
INLA: Integrated Nested Laplace Approximations
GMRF: Gaussian Random Markov Field
SSTCDapp: Spatial and Spatio-Temporal Count Data Application
UK: United Kingdom
